# Utilization of Multisensor Data Fusion for Magnetic Nondestructive Evaluation of Defects in Steel Elements under Various Operation Strategies

**DOI:** 10.3390/s18072091

**Published:** 2018-06-29

**Authors:** Grzegorz Psuj

**Affiliations:** Department of Electrical and Computer Engineering, Faculty of Electrical Engineering, West Pomeranian University of Technology, Szczecin, al. Piastow 17, 70-310 Szczecin, Poland; gpsuj@zut.edu.pl; Tel.: +48-91-449-4727

**Keywords:** nondestructive evaluation, magnetic flux leakage, multisource inspection, multisensor array, defect indication, steel elements, multisensor data fusion, signal data fusion, data fusion strategy

## Abstract

Increasing the number of inspection sources creates an opportunity to combine information in order to properly set the operation of the entire system, not only in terms of such factors as reliability, confidence, or accuracy, but inspection time as well. In this paper, a magnetic sensor-array-based nondestructive system was applied to inspect defects inside circular-shaped steel elements. The experiments were carried out for various sensor network strategies, followed by the fusion of multisensor data for each case. In order to combine the measurements, first data registration and then four algorithms based on spatial and transformed representations of sensor signals were applied. In the case of spatial representation, the data were combined using an algorithm operating directly on input signals, allowing pooling of information. To build the transformed representation, a multiresolution analysis based on the Laplacian pyramid was used. Finally, the quality of the obtained results was assessed. The details of algorithms are given and the results are presented and discussed. It is shown that the application of data fusion rules for magnetic multisensor inspection systems can result in the growth of reliability of proper identification and classification of defects in steel elements depending on the utilized configuration of the sensor network.

## 1. Introduction

There are currently two main aspects of newly introduced technological solutions: production and quality control. The technology used must meet high quality standards. By “quality”, we mean a high discrimination rate between possible material states, which, in consequence, allows highly effective correct identification of the actual condition and a high level of product safety. In practice, this involves the need to develop integrated inspection instrumentation coupling multisource systems and advanced methods of processing and data fusion [1,2,3,4,5,6,7,8,9,10,11,12,13,14].

According to the fact that most of the current structures are made of conductive and magnetic materials, the use of electromagnetic testing methods becomes a natural solution. The methods can be utilized to inspect surface and subsurface regions as well as deeper parts of the material for the detection of cracks or any inhomogeneity affecting the electromagnetic properties of the material. One of the most common electromagnetic nondestructive testing techniques used in the industry is magnetic flux leakage (MFL). The objective of the method is to detect the magnetic field over the inspected area which leaks from the material when a difference in magnetic conditions (permeability) appears, caused by a defect’s occurrence [1]. It finds its application in the inspection of any object with ferromagnetic properties. It is frequently utilized for the inspection of pipelines or tubes [1,4,5,6,7,8,15,16], train wheels and axles or rail tracks [9,17,18,19], steel wire ropes [10,11,20], storage tanks [15,21], or steel construction elements [2,12,22,23,24]. The method is suitable for detecting various types of surface, subsurface, and surface-breaking deeper defects in magnetic materials such as metal loss [2,10,11,20], fatigue damage cracks [1,2,3,5,8,9,16], pitting corrosion [1,2,5,6], or plastic deformation [1,15,24,25]. The relatively small dimensions of the detectors of leaking magnetic flux allow us to apply the method to examine both the external sides [1,2,3,4,5,9,10,11] of the tested objects and the internal sides such as the inner wall of a pipe or hollow axle [1,6,8,16,19]. One of the main advantages of the method is the lack of need for direct contact between the inspected element and the sensing device. Therefore, it can be applied even when the material is covered with paint or dust. One of the main challenges in the construction of measuring systems is the magnetization section. They can be built with a magnetizing coil generating an alternating (AC) or direct (DC) field or, alternatively, with a permanent magnet [1,4]. The AC source, due to the skin effect, is mostly used for surface condition monitoring. More frequently, the DC field is utilized, as it allows deeper inspection of the material. On the other hand, the sensing can be affected by some disturbance conditions [1,4,10]. Nevertheless, during the design and further inspection process, orientation of defects, which can possibly occur in the tested element in reference to the direction of the magnetizing field, should be considered. The sensitivity of the method strongly depends on the degree of disruption of the free flow of the magnetic field lines caused by anomalies in the material. Thus, detection of defects aligned parallel to the field lines path is restricted and can strongly affect the proper indication. One of the main limitations of the method is the scanning speed [1,9]. Utilization of a high testing speed can result in eddy currents affecting testing. Therefore, due to the simultaneous desire to increase the reliability and speed of conducted inspections, as well as to gain complementarity and completeness of the acquired examination information, there currently arises a strong need to design and build complex and distributed multiple homogenous or heterogeneous sources network for nondestructive control systems [3,12,17,18,19,22,23,25,26].

In case of industrial magnetic flux leakage inspection systems, the array of homogenous sensing elements has been most frequently utilized for inspection of steel wire rope [4,10,11] or the inner and outer wall of pipelines or any circle-shaped steel object [1,4,5,6]; however, usage for constriction condition monitoring has also been common [2,3,12,15]. In the majority of cases, the sensors were arranged in linear arrays [2,4,5,10,11,15] placed in the magnetic field and aligned parallel [2,4] or perpendicular [5,6,10,11] to the field lines, wrapping the surface of the material. During inspection, the arrays observed one or all three components of the magnetic field (tangential *x* and *y* and normal *z*). The scanning method was adjusted to the type of the tested element and the size of the device in reference to the size of the objects. Usually the array was moved linearly along the scanning direction [2,8,10,11]. In [11], a flexible GMR-type (giant magnetoresistive) sensor array fixed within a pair of saddle-type magnetizing coils was used for the detection of defects on the outer surface of steel track rope. The tested object was magnetized along the axial direction and the array was used to sense the axial component of leakage flux during linear movement. The system performance was validated using artificial defects (with length of few to few tens and depth of 2 to around 5 mm) made in the circumferential and axial directions. It showed some possibility for identifying the spatial information and a higher discrimination level for circumferentially aligned notches. A similar solution of array general construction was presented in [10]. Utilization of TMR-type (magnetoresistive tunnel) devices allowed high sensitivity even for defects with depth and length of 0.5 mm. However, the accuracy of presented solutions along with the scanning mode depends on the distance between two successive sensing elements and can result in misdetection of relatively small defects. A possible enhancement of the achieved effectiveness could be reached by a set of two shifted sections of sensor arrays following each other, like that demonstrated in [8]. In [9], the three sections, each comprising 16 Hall sensors, observing different components of a vector field and placed one by another were used for the detection of multiple cracks in steel elements of rail tracks. The monitoring of all three components allowed the evaluation of the shape and orientation of multiple cracks, but was not efficient in depth assessment. In the case of round-shaped examined elements, when the array length was less than the circumference of the object, the helical rotation mode was also applied in order to cover a greater inspection area [4,5,6] and preserve relatively high inspection speed. Nevertheless, in the majority of proposed solutions, in order to obtain a high inspection speed, the defects were detected each time only by a single sensor or a pair of neighbouring sensors. This could result in achieving restricted inspection resolution when a defect crosses the array line at the midpoint between two neighbouring sensing elements [10]. Furthermore, disrupted or incorrect indications provided by one element in the sensor array can also significantly limit the whole system’s defect detection ability and efficiency. Thus, the construction of the sensor array not only affects the examination speed but also plays a crucial role in the definition of the overall system’s performance.

Generally, under the concept of a multisensor inspection system, various aspects, such as risk of faulty indication, spatial coverage, imprecision, and uncertainty, should be considered [27]. In such cases a multisensor data fusion methodology appears as a natural solution of the combined problem. Such multisensor structures permit the adjustment of the sensing unit configuration under various terms including inspection area range and coverage of a single sensor, acquisition time, or reliability of results [27,28,29,30]. Thus, application of a data fusion procedure allows us to simultaneously balance the operation of the system in reference to the mentioned factors. Furthermore, it is possible to achieve greater robustness and reliability, greater spatial coverage and a gain of higher resolution, and an increase of confidence and decrease of uncertainty at the same time. Therefore, the choice of scanning mode, in the case of multisensor solutions, is an important factor that should be taken into account when assessing the relationship between system reliability and speed of inspection. According to [31], the sensors can operate under three major configurations: competitive, complementary, or cooperative. The array is running in competitive mode when each sensing element provides independent information about the same range of the inspected object and the combined information is building a redundant image. In fact, this configuration allows us, at the end, to reduce the risk of incorrect indication caused by failure of one sensor by minimizing its influence on the final result or even excluding it from building the image. In consequence, this might result in an increase in the reliability, accuracy, or confidence of the data. On the other side of configuration modes, the complementary one arises. Utilization of this strategy means that each sensor is monitoring a different selected part of the examined object and collected information by all elements is combined to provide a broader image of the tested material. Finally, the cooperative mode refers to usage of a set of sensors to deliver one different aspect of the same object and create, in that manner, a more comprehensive image of the inspected material. The described categories are not mutually exclusive and a set of two sensors could be operating within the complementary, competitive, or cooperative mode in a different inspection range. Therefore, taking into account the specific feature of operation of the measurement system to be optimized, it is possible to apply various strategies. Regardless of the final configuration of the sensor array, the use of multisensor collection and data fusion allows us to gain an improvement in the general system performance.

Some initial consideration of multisensor data fusion under the competitive mode was previously presented in [26]. In the current paper, taking into consideration all described aspects, multisource data fusion is carried out on multisensor array data obtained during inspection of defects in a circular-shaped tube sample under different terms. Several strategies of sensor array operation are examined and the resulting performance is evaluated. Under each considered configuration, information gathered from the sensor array is fused using different fusion algorithms. The aim of the process was to gain a higher quality of inspection results and thus increase the possibility of obtaining correct indication and evaluation of defects, balancing reliability and inspection time. During the integration of data, several aspects were considered. First, multisensor data registration is carried out. Then, the applied fusion algorithms are described and the obtained results discussed. Finally, the performance is evaluated and the conclusions are presented.

## 2. Multisensor Data Source and Fusion Strategies

**Figure 1:**
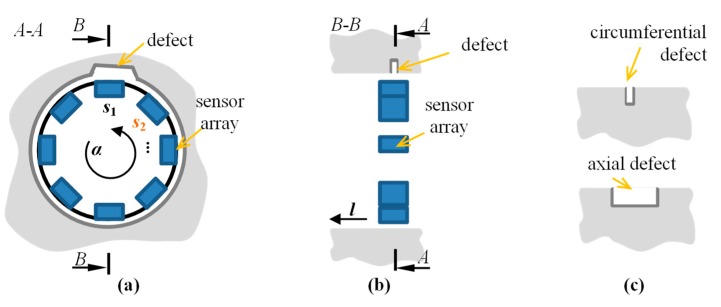
Visualization of multisensor array arrangement inside a steel sample with depicted circumferential *α* and axial *l* directions: cross sections in (**a**) circumferential A-A and (**b**) axial B-B planes of the sensor array; (**c**) cross section in the axial plane of two types of examined defects.

**Figure 2:**
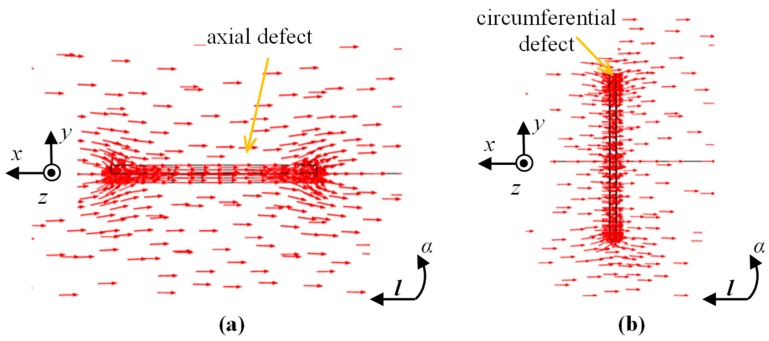
Visualization of magnetic leakage vectors over axial (**a**) and circumferential (**b**) types of defect with depicted scanning circumferential *α* and axial *l* directions and sensing *x*, *y*, and *z* directions.

In order to process the experiments, the magnetic sensor array data were used. The data were acquired during inspection of a circular-shaped tube magnetic steel sample with six artificial defects: *d*_1_–*d*_6_ (Figure 1). All were made on the internal wall of the sample using the EDM (Electro Discharged Machined) method in two main orientations with reference to the axis of symmetry (Figure 1c): in the axial (*d*_1_, *d*_3_, and *d*_5_) and circumferential directions (*d*_2_, *d*_4_, and *d*_6_). The defects had the same length of 5 mm and width of 0.5 mm while the depths varied: 0.5 mm for *d*_3_ and *d*_4_, 1 mm for *d*_1_ and *d*_2_, and 2 mm for *d*_5_ and *d*_6_. The data were acquired under the DC magnetic field conditions. Before scanning, the internal surface of the sample was magnetized in the axial direction, by moving along its axis *l* the permanent magnet with magnetic poles placed in the axial direction. A visualization of the course of the obtained leakage field vectors over the surface of the inner wall, in the vicinity of defects located axially and circumferentially, is shown in Figure 2. It can be noticed that the circumferential defects are located perpendicularly to the direction of the field lines, hence causing greater disturbance of the field. As a result, a higher indication value of this type of defect is achieved. The residual flux leakage field is then measured by an array of 3-axis AMR-type (anisotropic magnetoresistive) sensors (Figure 1a,b). The sensors were fitted within a hole of 30 mm diameter in equally spaced positions located every 45 degrees on the circumference, which resulted in having 8 sensors in an array. Each sensor was measuring three components of magnetic field: two tangential to the surface (at a point) of the sample, along *V*_x_ and across *V*_y_ the magnetizing stream lines, and the normal *V*_z_.

**Figure 3:**
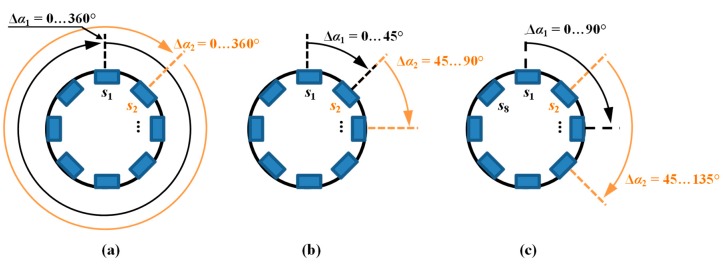
Presentation of implementation of various multisensor data fusion strategies: (**a**) competitive; (**b**) complementary; and (**c**) cooperative data.

**Figure 4:**
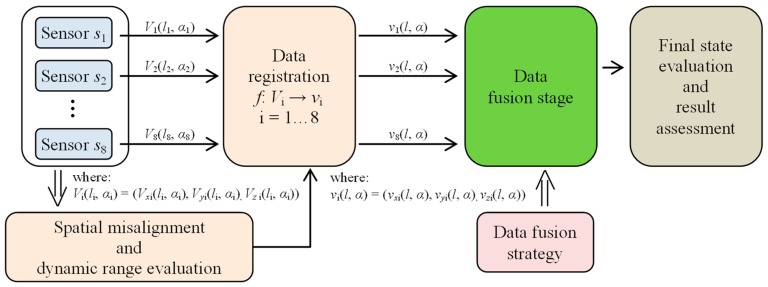
The general overview of the successive stages of the defect evaluation process.

**Figure 5:**
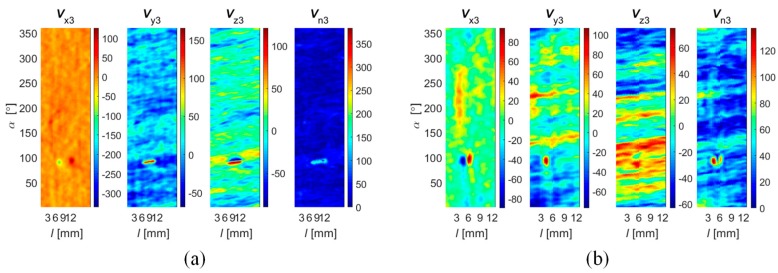
Distributions of *V*_x_, *V*_y_, and *V*_z_, and norm *V*_n_ values of vector field sensed by selected single sensor (*s*_3_) obtained for (**a**) the 2 mm depth axial defect (*d*_5_) and (**b**) the 0.5 mm depth circumferential defect (*d*_4_) over 2-D scanning area (*l*, *α*); unit: 100 equals 0.4 G.

During the data collection, the array was first moved by linear steps Δ*l* along the axial direction *l* and data acquisition was then carried out during rotation (circumferential direction *α*) by a given angle Δ*α* (Figure 1a). In order to evaluate the performance under different operation strategies, the range of the rotation angle Δ*α* was changed during the experiments from 45 up to 360 degrees in steps of 45 degrees. This enabled one to obtain a different spatial range of data for each sensor and, in consequence, operation of the array under competitive (Δ*α* equal to 360°), cooperative, or complementary (Δ*α* equal to 45°) conditions. When the rotation angle was equal to 360 degrees, each sensor was moved over the whole inspection area (full rotation) and thus provided data corresponding to other sensors’ areas, which was shifted from sensor to sensor by 45 degrees in the angular direction (Figure 3a). On the opposite side (in terms of the scanning angular range) is the case of rotating the sensor array by only 45 degrees (Figure 3b). In this situation, each sensor observes only a selected—not covered by any other sensor—part of the examined sample’s area. This configuration results in the complementary mode of the sensor array. When the rotation angle was larger than 45 and less than 360 degrees, the multisensor unit could operate in cooperative mode, allowing both competition and cooperation of the sensor set. In Figure 3c, a schematic visualization of the array configuration obtained for a rotation angle equal to 90 degrees is shown. In this example, sensors *s*_1_ and *s*_2_ cover the angular areas defined by Δ*α*_1_ and Δ*α*_2_ ranging from 0 to 90 and from 45 to 135 degrees, respectively. Considering only those two sensors’ data for angles lower than 45 and greater than 90 degrees, the array was operating in complementary mode. In the middle range (45–90 degrees), the competitive strategy could be applied. Nevertheless, no matter what the applied Δ*α* range, the scanning resolution was always equal to 0.1 mm and 0.1 degree. A detailed description of the construction of the multisensor array used as well as the sample can be found in [19]. The diagram of the whole process for defect evaluation, applied regardless of the utilized operation mode, is presented in Figure 4. First, the sensors’ data were matched and transformed into the same representation space and output value dynamic range (data registration stage). Then, data fusion was processed and, finally, the evaluation of the obtained results was carried out. The selected results of measurements of all three components measured by one sensor obtained for defect *d*_5_ and *d*_4_ with respective depths of 2 mm and 0.5 mm are presented in Figure 5. Taking into consideration the depths, the defects represent two extreme detection cases: the easiest and the hardest, respectively. Each measured field component allows us to gain different information about the defect, such as the length, width, and depth [12,18,19,23]. However, it can be noticed that, especially for shallower defects, beside the visible indication of signals in the faulty area, in other parts, the signal level is also high; this can result in misclassification of the actual state. Therefore, the need for data fusion arises.

## 3. Spatial Alignment Evaluation and Data Registration

**Figure 6:**
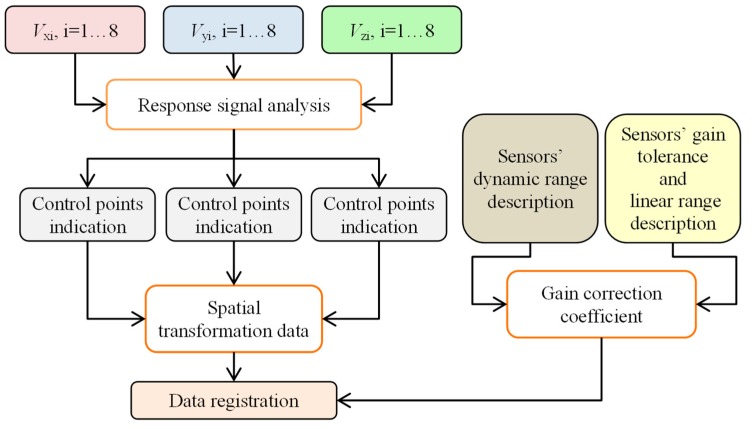
The block diagram of the data registration process.

**Figure 7:**
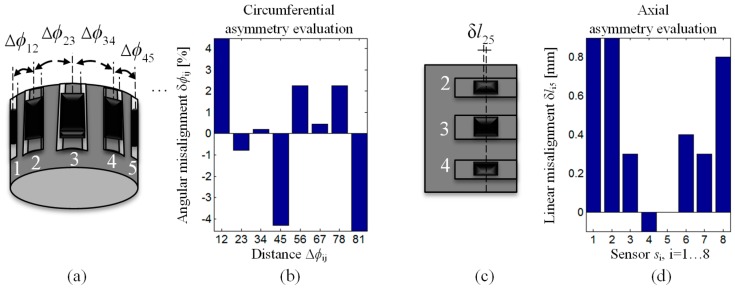
Spatial alignment evaluation of the transducer’s sensors: (**a**) view of the sensor array with depicted angular distances Δ*ϕ* between two successive sensors,; (**b**) results of the angular misalignment δ*ϕ* estimation; (**c**) visualization of linear misalignment δ*l* estimation as a difference between center locations in linear positions of given sensor *s_i_*, *i* = 1, 2, 3, 4, 6, 7, 8, and the sensor *s*_5_, and (**d**) results of the sensor linear misalignment δ*l* estimation.

Before processing the fusion, the data registration procedure was carried out. The functional block diagram of the registration algorithm is presented in Figure 6. First, in order to transform acquired signals into a common coordinate system, the estimation of the angular δ*ϕ* and linear δ*l* misalignment of each sensor in the array must be carried out. For that need, the measurements for defect *d*_6_ (allowing archiving of the highest level of signal corresponding to the defect) were processed under the competitive mode. In consequence, data were acquired in the full angular range for all 8 sensors. Then, for the misalignment evaluation, the analysis of sensor vectors *V*(*l*, *α*) *=* (*V*_x_(*l*, *α*), *V*_y_(*l*, *α*), *V*_z_(*l*, *α*)) was carried out. First, based on the location of characteristic values of the components’ signals in the vicinity of the defect *d*_6_, the control points *cp* were determined individually for each sensor. Then, the spatial relationship between defined *cp* for each sensor in reference to the ones for sensor *s*_5_ was analyzed. The *s*_5_ sensor was chosen as it was placed in the middle of the multisensor array, so for sensors of number lower than 5, the shifting angle was positive and for ones greater than 5, it was negative. The results of the angular misalignment evaluation are presented in Figure 7. According to the results, it can be noticed that the asymmetry of the sensor allocation cannot be neglected. The highest angular misalignment is around 4% which corresponds to 1.8° while the linear misalignment is 1 mm. Taking into consideration the diameter (close to 29.5 mm) of the sensor array, the angular misalignment δ*ϕ* can be recalculated (multiplying by (π/180) R, where R is the radius) and expressed in corresponding mm scale. This allowed the comparison of the allocation error with the dimensions of possible defects. The estimated value of misalignment recalculated to arc distance was less than 0.5 mm, which can be assessed as fairly acceptable for industrial applications.

**Figure 8:**
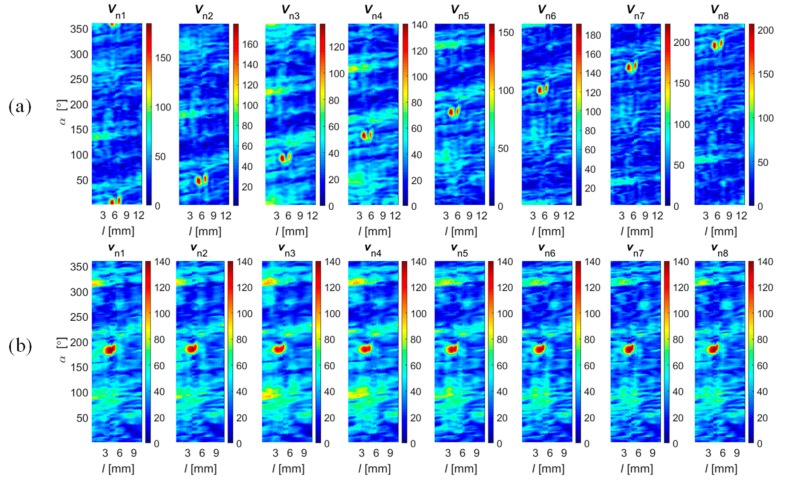
Distributions of field *V*_n_ and *v*_n_ norm sensed by successive sensors obtained for 0.5 mm depth circumferential defect (*d*_4_) over 2-D scanning area (*l*, *α*): (**a**) before and (**b**) after the registration process; unit: 100 equals 0.4 G.

**Figure 9:**
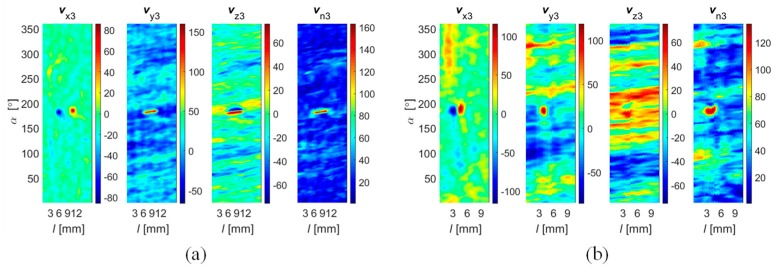
Distributions of registered *v*_x_, *v*_y_, and *v*_z_, and norm *v*_n_ values of vector field sensed by a selected single sensor (*s*_3_) obtained for (**a**) a 2 mm depth axial defect (*d*_5_) and (**b**) a 0.5 mm depth circumferential one (*d*_4_) over 2-D scanning area (*l*, *α*); unit: 100 equals to 0.4 G.

**Figure 10:**
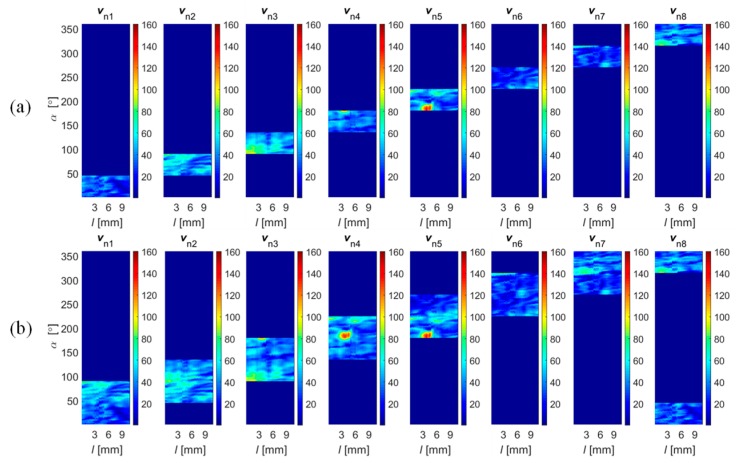
Distributions of field *V*_n_ norm sensed by successive sensors obtained for a 0.5 mm depth circumferential defect (*d*_4_) over 2-D scanning area (*l*, *α*) for an angular range of each sensor equal to (**a**) 45° and (**b**) 90°; unit: 100 equals to 0.4 G.

In the second step, the sensitivity range of sensors in the array was estimated. During this stage, not only the gain and the offset but also the difference in distance between each sensor and the surface of the material should be taken into account. Considering the gain tolerance and linearity ranges of the sensors and the experimental results, the calibration coefficients were computed, allowing one to obtain similar output signal dynamic ranges for each sensor under the same measuring conditions. Finally, the data transformation into common representation was carried out. The selected results (obtained for all sensors) of vector norm values obtained for all sensors are presented in Figure 8, and those for a single sensor for all field components and the norm value are in Figure 9. It can be seen (Figure 8) that the applied process allowed one to obtain corresponding spatial as well as sensitivity ranges for each sensor. The spatial and sensitivity transformation information was also utilized in the case of data acquired under complementary and cooperative modes. The result of the registration process obtained for Δ*α* values equal to 45 and 90 degrees are presented in Figure 10. Finally, the achieved registered signals of all components were then used as input for the data fusion algorithms.

## 4. Data Fusion Algorithms

**Figure 11:**
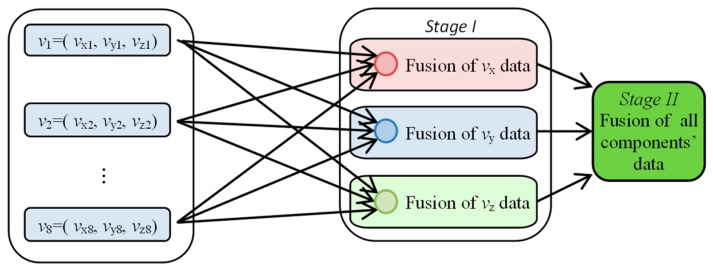
Block diagram of the general data fusion scheme.

The utilization of a multisensor array gives the advantage of limiting the influence of unwanted distortions on the observation of any single sensor, thus affecting the efficiency of proper defect indication [30,31,32,33,34,35,36,37]. In order to combine information provided by all sensors, data fusion algorithms were utilized. The block diagram of the general data fusion scheme is presented in Figure 11. First, the registered vector *v* data of all sensors were fused. The procedure was carried out separately for each component of *v* resulting in common representations of *v*_x_, *v*_y_, and *v*_z_ components (Stage I of the data fusion algorithm). Then, taking into consideration that each component can provide one with different complementary data about the detected defect (which can enhance the efficiency of defect indication), the final integration was performed by computing the norm value of all components’ fused distributions (Stage II of the data fusion algorithm).

**Figure 12:**
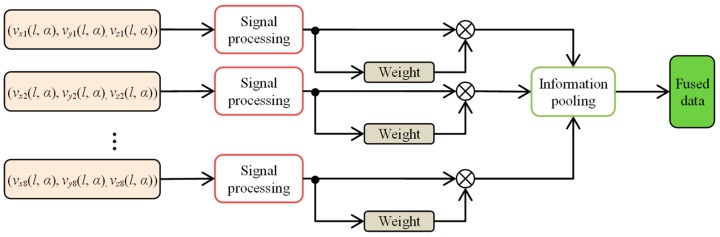
Block diagram of the data fusion process: Stage I.

In order to process the Stage I algorithms (Figure 12), spatial and transformed representations [31,32,33,35] of sensors’ signals allowing pooling of information were applied. The spatial framework can be expressed by

*v*_df_(*l*, *α*) = *f*_df_ (*v*_1_(*l*, *α*), *v*_2_(*l*, *α*), _…_, *v*_8_(*l*, *α*)) (1)

while the transformed one can be given in the form

*v*_df_(*l*, *α*) = **T**^−1^{*f*_df_ (**T**{*v*_1_(*l*, *α*)}, **T**{*v*_2_(*l*, *α*)}, …, **T**{*v*_8_(*l*, *α*)})} (2)

where *f*_df_, T, and T^−1^ respectively denote data fusion, transform, and inverse transform operators, and *v*_df_ is the fused distribution of the given component. Finally, in order to carry out the integration, four fusion rules were introduced considering spatial vectors, spatial information, and multiresolution representations of multisensor data.

### 4.1. Spatial-Vector-Based Data Fusion (DF_vec_ Algorithm)

Taking into consideration that the transducer consists of sensors of the same type, each sensor should acquire similar observations in reference to common global position. Therefore, a signal’s representation in terms of specific global position on the measuring area is represented by a common vector for all sensors defined in 8-dimensional feature space as

*v*(*l*, *α*) = (*v*_1_(*l*, *α*), *v*_2_(*l*, *α*), _…_, *v*_8_(*l*, *α*)). (3)

If any sensor or group of sensors indicates a defect, the length of the vector would change. The rate of variation grows with the number of sensors that provide the indication information. As a result, the common representation spatial vector can be defined as

(4) $$\;v_{\mathrm{df}}\left(l,\;\alpha \right)=\sqrt{v_{1}{\left(l,\;\alpha \right)}^{2}\;+\;\ldots \;+\;v_{8}{\left(l,\;\alpha \right)}^{2}}.$$

Finally, results of spatial vectors for three components were obtained: *v*_xdf_(*l*, *α*), *v*_ydf_(*l*, *α*), and *v*_zdf_(*l*, *α*).

### 4.2. Direct Spatial Multisensor Data Fusion

In the direct spatial fusion, the sensors’ input signals are integrated using localized spatial features. In this process, three aspects of multisensor perception for state evaluation can be considered: conjunctive, disjunctive, and compromise [30].

#### 4.2.1. Conjunctive and Compromise Concepts (*DF*_SF_ Algorithm)

The first concept leads to building a fused representation including all input information, regardless of the information importance of each source. This can be simply expressed by

(5) $$\;v_{\mathrm{df}}\left(l,\;\alpha \right)=\sum v_{n}\left(l,\;\alpha \right).$$

The first approach can be complemented by taking into account the significance of information from individual sources, also called “building a compromise”. According to Equation (5), the expression can be defined as

(6) $$\;v_{\mathrm{df}}\left(l,\;\alpha \right)\;=\frac{1}{N}\overset{N}{\underset{i=1}{\sum }}w_{i}v_{i}\left(l,\;\alpha \right)$$

where *w*_i_ stands for the weight of the given ith source referring to its information content (which can be described by various measures allowing quantification of data) and *N* is the number of sources. This definition in terms of performance evaluation can be also understood as a linear opinion pool (LOP) approach [32]. This strategy is commonly utilized. The weighted sum of data gathered by similar sources is frequently utilized in signal fusion algorithms [33,34,35,36]. The main objective is to build a common representation enhancing the overall information level. One of the most straightforward fusion operators of this type is simple superposition by averaging of the inputs. Then, all source weights are equal. In other cases, the most important aspect is to obtain the weight coefficient representing the information level supplied by the specific source. The applied coefficient computation algorithm can be based on classical statistics (e.g., variance or standard deviation) or on analysis of information content based on, e.g., principal component analysis (PCA) [34] or spatial frequency (SF) analysis [37,38]. In this paper, the signal processing was carried out for each sensor data and the overall level of information acquired by a specific sensor was estimated. For that need, a spatial frequency *SF*-analysis-based algorithm was utilized in order to compute the weights corresponding to the information level provided by input sources. In order to process the calculation, first the row (*RF*) and column (*CF*) frequencies and then *SF* were computed based on the following expressions [32,34]:

(7) $$\;RF=\;\sqrt{\frac{1}{PQ}\overset{P-1}{\underset{p=1}{\sum }}\overset{Q-1}{\underset{q=2}{\sum }}{\left[v\left(p,q\right)-v\left(p,q-1\right)\right]}^{2}}$$

(8) $$\;CF=\;\sqrt{\frac{1}{PQ}\overset{P-1}{\underset{p=1}{\sum }}\overset{Q-1}{\underset{q=2}{\sum }}{\left[v\left(p,q\right)-v\left(p-1,q\right)\right]}^{2}}$$

(9) $$\;SF=\sqrt{RF^{2}+CF^{2}}$$

(10) $$\;w_{i}=\frac{SF_{i}}{\sum SF}$$

where *P* and *Q* refer to number of rows and columns of analyzed data *v*, *i* = 1, …, *N*, and *N* is the number of data sources. The obtained weights *w*_i_ were then used to obtain the fused data for each component (*DF*_SF_ algorithm).

#### 4.2.2. Disjunctive Concept (*DF*_IOP_ Algorithm)

The aim of the second concept is to maintain common information in the fused data, i.e., that appearing in all the component sources, and to suppress mutually different information at the same time. In this case, the expression can be given as

(11) $$\;v_{df}\left(l,\;\alpha \right)=\prod v_{n}\left(l,\alpha \right)$$

Considering the described properties and assuming that the information conditioned on the measurement set is independent, this strategy can be defined also as an independent opinion pool (IOP). This concept was utilized in this paper to depict the areas of high confidence of indication (*DF*_IOP_ algorithm).

### 4.3. Multiresolution-Analysis-Based Data Fusion (DF_PYR_ Algorithm)

Multiresolution or multiscale analysis (MRA) is a well-known transformation framework in signal (or image) fusion processing. Its main idea is to decompose the input data into successive subsets (layers) of different frequency and resolution representations [33,34,39]. In reference to Equation (2), MRA-based data fusion concerns two aspects: first is the decomposition algorithm, while the second one is the fusion rule [28,35,39]. Commonly utilized transformations are based on wavelet, contourlet, or, recently, shearlet decomposition. Resulting subsets represent details of input sources at different scales and orientations. The other type of processing technique is pyramid decomposition. In this case, each layer is filtered and downsampled by a factor of 2 from the representation of a previous one. The decomposition process can be applied utilizing different transformation algorithms such as contrast, Gaussian, or Laplacian filtering [28,33,40]. The main idea of the multiscale fusion scheme is to combine the information at different detail levels in order to preserve the information of different scales. Then, the multiresolution integrated data is reconstructed to obtain the final fusion result.

In this paper, a Laplacian pyramid was utilized to achieve the multilayer representation of data for each sensor [32,38,39]. In order to process the Laplacian pyramid transformation, first, the original data *vpyr*{0} were decomposed into a 5-layer set *vpyr* containing sequentially reduced representations. The reduction operation is a recursive (level-to-level) averaging process carried out according to the following expression:

(12) $$\;vpyr\left\{m\right\}\left(p,q\right)=\overset{2}{\underset{a=-2}{\sum }}\overset{2}{\underset{b=-2}{\sum }}w\left(a,b\right)vpyr\left\{m-1\right\}\left(2p+a,2q+b\right)$$

where *m* refers to a given layer and is within the range 0 < *m* < 5, *p* and *q* are indices of rows and columns of a given layer defined within the range 0 ≤ *p* < *P*_m_, 0 ≤ *q* < *Q*_m_, and *P*_m_ and *Q*_m_ are the number of rows and columns of the *m*th-level representation. The utilized weight *w* kernel width was equal to 5, which allows one to obtain balance between the filtering conditions and computational time. The kernel is defined by the set {0.25-*cw*/2, 0.25, *cw*, 0.25, 0.25-*cw*/2}, where *cw* is a center weight. The inverse operation (expansion) at a given level is obtained by interpolating new values between the given ones according to the expression

(13) $$\;vpyr\left\{m+1\right\}\left(p,q\right)=\;4\overset{2}{\underset{a=-2}{\sum }}\overset{2}{\underset{b=-2}{\sum }}w\left(a,\;b\right)vpyr\left\{m\right\}\left(\frac{p-a}{2},\frac{q-b}{2}\right).$$

After decomposition, the Laplacian pyramid set L_vpyr_{0}, …, L_vpyr_{4} is calculated as a sequence which each level is the difference L_vpyr_{*m*} = *vpyr*{*m*} − Expand (*vpyr*{*m* + 1}). The original data distribution can be achieved by expanding and summing operations of the L_vpyr_ set_._

**Figure 13:**
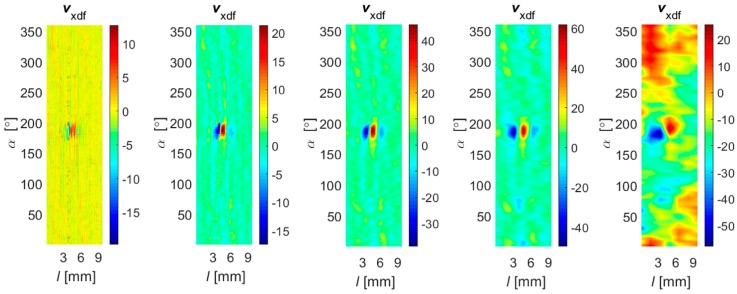
A visualization of exemplary results of successive levels of Laplacian pyramid decomposition obtained for *v*_x_ of a selected sensor.

An exemplary view of representations obtained for successive layers of a selected sensor component *x* distribution (the resolution is decreasing from the left-hand side to the right-hand side) is presented in Figure 13. One can notice that the information content about the inspected defect is the highest for the three middle layers. It is clear that the distributions on both the first and the last layer provide ambiguous data, which may have a significant influence on further identification processes. Considering that the characteristic of MFL inspection signals in the vicinity of the defects rather represents the middle spatial frequency range, the highest signal representation could be neglected. Similarly, the lowest one can present not only information about the defect but also a high level of low-frequency background noise. Finally, the fusion was processed for the middle representation layers (as the first and the last layers of the representations were filtered out). The fusion at a specific layer was performed under the LOP strategy described earlier by computing the average signal, and the reconstruction process was then carried out.

## 5. Data Fusion Results

**Figure 14:**
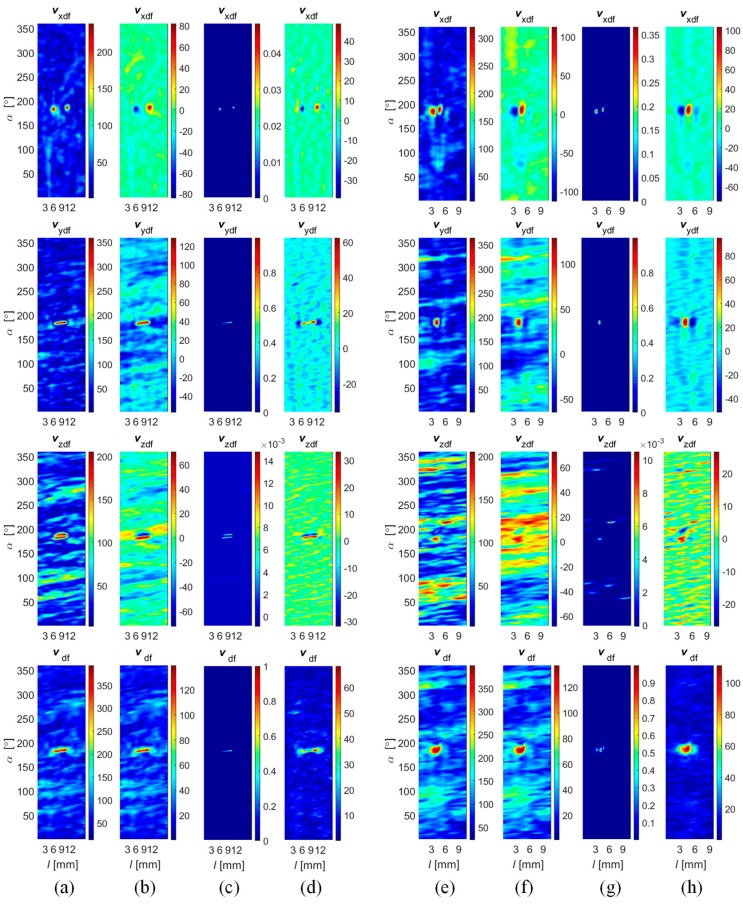
Results of Stages I (*v*_xdf_, *v_y_*_df_ and *v*_zdf_) and II (*v*_ndf_) of data fusion obtained for axial defect *d*_5_ with 2 mm depth using (**a**) *DF*_vec_; (**b**) *DF*_SF_; (**c**) *DF*_IOP_; and (**d**) *DF*_PYR_ algorithms; and for a circumferential defect *d*_4_ with 0.5 mm depth using (**e**) *DF*_vec_; (**f**) *DF*_SF_; (**g**) *DF*_IOP_; and (**h**) *DF*_PYR_ algorithms.

**Figure 15:**
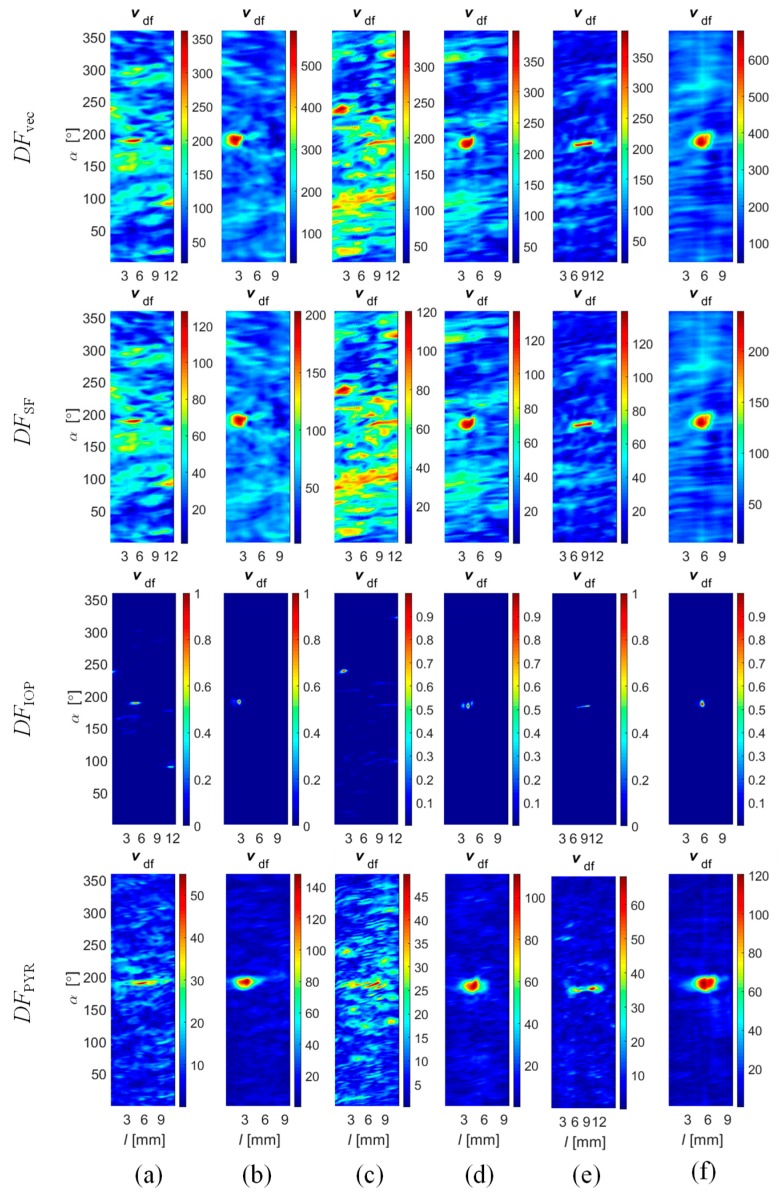
Results of Stage II (*v*_ndf_) of data fusion obtained for successive defects: 1 mm depth axial *d*_1_ (**a**) and circumferential *d*_2_ (**b**); 0.5 mm depth axial *d*_3_ (**c**) and circumferential *d*_4_ (**d**); and 2 mm depth axial *d*_5_ (**e**) and circumferential *d*_6_ (**f**).

**Figure 16:**
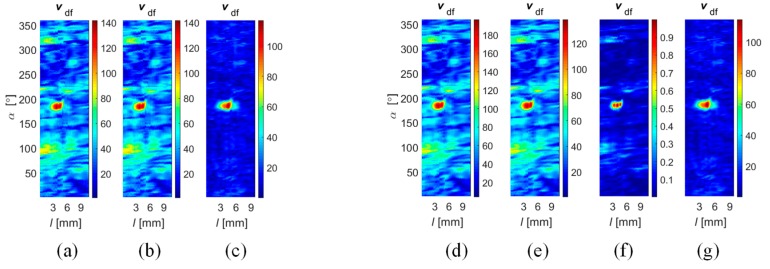
Results of Stage II (*v*_ndf_) of data fusion obtained for the 0.5 mm circumferential *d*_4_ defect achieved for angular range of each sensor equal to 45° and using algorithms (**a**) *DF*_vec_; (**b**) *DF*_SF_; and (**c**) *DF*_PYR_; and for angular range equal to 90° and using algorithms (**d**) *DF*_vec_; (**e**) *DF*_SF_; (**f**) *DF*_IOP_, and (**g**) *DF*_PYR_.

The selected results obtained during the fusion process at both stages (Figure 11, results of Stage I: *v*_xdf_, *v*_ydf_, *v*_zdf_ and Stage II: *v*_df_) carried out using all four fusion rules are presented in Figure 14, Figure 15 and Figure 16. First, the results obtained under competitive operation of the multisensor array were analyzed (Figure 14 and Figure 15). Considering the outputs of *DF*_vec_ and *DF*_SF_, one can see that both show relatively similar information content (similar characteristics of distributions). The major difference between the rules concerns the results’ dynamic range, as a significant gain of *DF*_vec_ distribution values in reference to *DF*_SF_ ones can be noticed. Thus, it can be stated that *DF*_vec_ allows us to obtain qualitatively slightly better discrimination and defect indication rate, which can be observed especially for shallower defects (Figure 14e).

Generally, on the basis of the presented results, the greatest growth of difference between the defect response signal and the background signal is qualitatively observed for the *DF*_IOP_ algorithm. It can be noticed that the clear indication of flaw edges is possible. This can be utilized in further processing of the defect identification process and lead to the enhancement of proper indication confidence and accuracy. However, it should be noted that in the case of a lower level of defect response signals (such as that observed for shallower axial defects), the background signals can be enhanced by a similar factor and the defect detection process can lead to an increase in the probability of false alarm (PFA) as well (case of *d*_1_ and *d*_3_, Figure 15a,c). In reference to this conclusion, the highest reduction of the influence of background signals on results was achieved for the *DF*_PYR_ rule. Once again, the greatest advantage can be seen for shallower defects. However, for each defect it is possible to notice a significant reduction of noise background when comparing with the *DF*_SF_ and *DF*_vec_ results. 

According to the utilized magnetization conditions, a worse discrimination ability was obtained for axially aligned defects (*d*_1_, *d*_3_, and *d*_5_), as they cause much lower disruption of the free flow of magnetic field in the examined element (Figure 15a,c,e). Nevertheless, also for this type of defect, it was clearly visible that there was an advantage in using the *DF*_PYR_ fusion scheme.

However, before further defect assessment procedures, the orientation of the defect should be evaluated first, as its alignment with respect to the magnetizing stream lines strongly affects the level of leaking magnetic flux and, thus, the indication process. The relationship between the depth and the signal levels is different for axial and circumferential defects.

In order to evaluate the efficiency of the fusion rule under different operation strategies, experiments were undertaken for different values of rotation angle Δ*α*. Selected results obtained for Δ*α* equal to 45 and 90 degrees during the inspection of defect *d*_4_ are presented in Figure 16. In the first case, the multisensor array was working in the complementary mode, as each sensor was providing unique data about a selected area of the examined object. Therefore, the application of algorithms *DF*_vec_ and *DF*_SF_ leads to the same results due to the processing of the fusion procedures, for each 45 degrees, from only one source of data (Figure 16a,b). On the same basis, the IOP strategy was not possible to carry out as it requires at least two sources for each part of the inspected area. In that situation, the greatest advantage can be observed in the case of the *DF*_PYR_ fusion algorithm. In the case of experiments carried out for Δ*α* equal to 90 degrees, all fusion schemes were carried out (Figure 16d–g). Even though the source number was increased only to two, a slight reduction of the background signal level could be noticed. From the qualitative evaluation of results obtained for both strategies, it can be seen that the level of the background signal is locally higher (particularly in the noise areas); this can be especially observed for the *DF*_vec_ and *DF*_SF_ results.

## 6. Evaluation of Data Fusion Results

In order to quantitatively evaluate the performance of the applied data fusion schemes, two quality factors were computed: entropy *E* and signal-to-noise ratio *SNR* [32,34,38]. The entropy is defined as

(14) $$\;E=-\overset{K}{\underset{k=1}{\sum }}Hv\left(k\right)\log_{2}Hv\left(k\right)$$

where *Hv* is the normalized histogram of distribution *v* and *K* is the number of histogram bins. It is expressing the information contribution of data and is related with the frequency of change in *v*. It is high for signals with high information content. However, it is also strongly correlated with the noise level and unwanted fluctuation of the signal. In the particular nondestructive application presented in this paper, the defect region covers only a relatively small part of the inspected area. Thus, it is expected that the signal variation would occur only in that region. Therefore, it can be understood that the lower the entropy value is, the better the performance of the fusion scheme. The *SNR* metric measures the ratio between useful (signal in defected area *v*_signal_) and unwanted signals (background signal level *v*_noise_):

(15) $$\;SNR\;=20\log_{10}\frac{v_{\mathrm{signal}}}{v_{\mathrm{noise}}}.$$

The *SNR* was calculated based on two areas: the area where the defect occurred and an area without the defect. *v*_signal_ and *v*_noise_ represent the mean values of signals in the areas where the defect occurred and without the defect, respectively. Both were obtained utilizing the thresholding operation, where the threshold value was calculated based on the values range in the defected area. In this case, a higher *SNR* value means that the discrimination ability grows.

The achieved values obtained for single-source signals and for the fusion algorithms under the competitive strategy for norm value (Stage II: *E*, *SNR*) are presented in Table 1 and Table 2.

**Figure 17:**
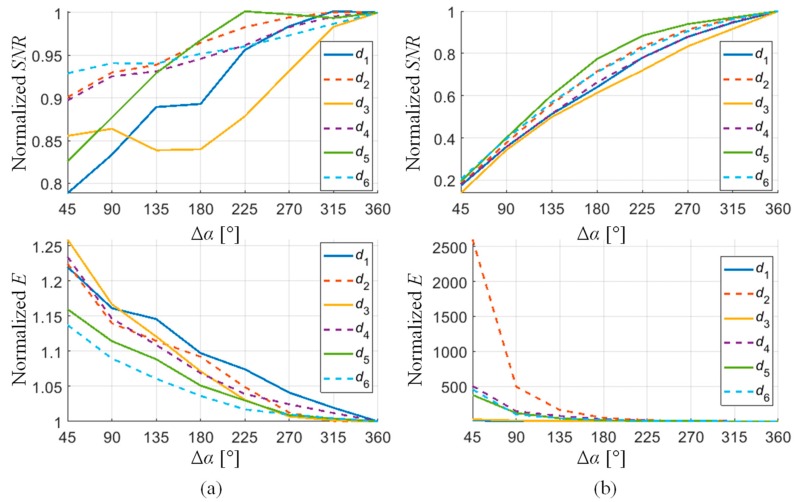
Changes of *SNR* and *E* coefficient levels in response to the increase of the angular measuring range of a single sensor from 45° (complementary data) to 360° (redundant data): computed for *DF*_PYR_ (**a**) and *DF*_IOP_ (**b**).

The obtained measures of *E* and *SNR* confirmed that by carrying out the applied data fusion schemes, an increase in the signal quality can be achieved. One can see a quantitative gain of information when comparing the results of raw signals *v* and the fused *v*_df_ ones of the second stage. The lowest change in *E* (drop of value) and SNR (increase in value) could be noticed in the case of *DF*_vec_ and *DF*_SF_ fusion schemes. Significant variation of values is visible for *DF*_PYR_ and the greatest can be observed for *DF*_IOP_. However, it should be underlined that even though the *DF*_IOP_ scheme results in significant reduction of noisy content, it can also affect the signal level of the defect area. As the algorithm allows us to maintain the common part of the data in the fused result and to suppress mutually different parts at the same time, both background noise and the defect signal level and area can be reduced. From that point of view, the utilization of the *DF*_PYR_ rule seems to be the most advantageous. In order to assess the change of the performance with respect to the operation strategy, the *E* and *SNR* were calculated for the results of *DF*_PYR_ and *DF*_IOP_ algorithms achieved for different ranges of rotation angle Δ*α*. For Δ*α* equal to 360 degrees, the sensors were working in competitive mode, while for 45 degrees, they were in complementary mode. All middle ranges resulted in usage of the cooperation strategy. By successively adding 45 degrees to the previous range of rotation angle, the number of sensors covering each 1/8 of the full rotation area is also increased by one. The results were normalized to the value obtained for the competitive mode and presented in the form of the plot in Figure 17. The obtained results confirmed that utilization of a greater number of sensors for processing the fusion scheme allows one to obtain a higher quality (confirmed by better levels of both evaluation factors); however, a higher gain in effectiveness can be noticed for harder indication conditions such as in the case of axial defects. It can be noticed that only in the case of the *d*_3_ defect is there a slight decrease of the *SNR* value for lower angular range. The defect *d*_3_ is an axial-type defect, which under the utilized magnetizing conditions is harder to detect than the circumferential one. Additionally, the defect is only 0.5 mm deep; therefore, it is the worst case (hardest to detect). The examined defect is close to the detection limit of the system as the defect signal is significantly lower in comparison to other cases. Therefore, the observed background signal can influence the ability of the system and affect the performance at a much higher rate. As a result, when only few sensors are used during inspection of a given part, even a slight decrease of *SNR* can be obtained. Thus, only when more than 4 sensors are used to scan the given angular range of the sample can the monotonic rise in the *SNR* be noticed.

In the procedure of performance evaluation of data fusion schemes, additional aspects can be considered. The application of data fusion allows one to simultaneously balance the operation of the system in reference to the factors related to inspection area range and coverage of a single sensor, reliability of results, and acquisition time. Due to the limitations in the scope of the scanning speed increment (at higher scanning speed, the eddy current could strongly affect the results), the multisensor array could be utilized simultaneously for decreasing the scanning speed for minimization of the eddy current effect and preserving the inspection time at least at a similar duration level. Another aspect is the dimensions of defects that might occur in the material, which the system should be able to indicate. Then, the scanning resolution should be at least similar to those dimensions. In this paper, the sampling frequency could be adjusted within the range of 40–400 Hz. The inspection time contains two periods: first, the scanning combined with data acquisition, and the period for resetting a new linear position. Taking into consideration the defect dimensions on the surface of the examined sample (5 mm length and around 0.5 mm width), the inspection time, when using all 8 sensors to perform the 45° scan (resulting in angular rotation range of 45°), could be increased from around 4 times for the highest sampling frequency to around 7 times for the lowest one in comparison to a 360° scan.

**Figure 18:**
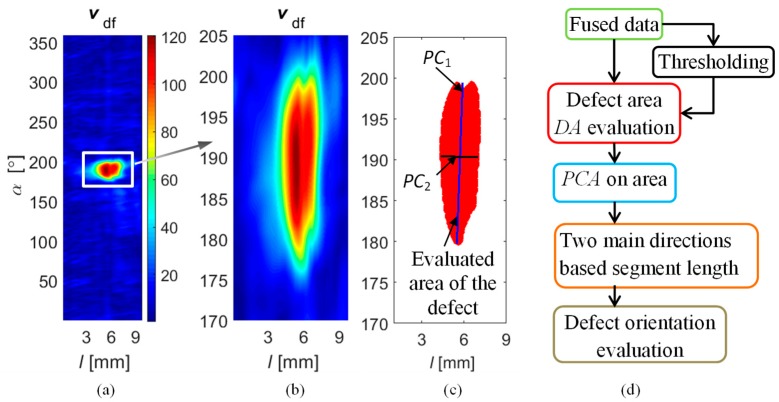
Results of successive stages of final defect identification procedure: fused results (**a**); zoomed defect area (**b**); indication of evaluated defect area together with the two main axes of area variation (**c**); block diagram of the successive steps of the defect orientation procedure (**d**).

In order to validate the information content enhanced by the applied fusion rules, an additional experiment was carried out. The aim was to assess the ability of the obtained results to correctly indicate the occurrence of defects. As it was described earlier, the defects’ orientation with respect to the direction of the magnetizing field plays an important role in the proper defect characterization process. Therefore, an automatic procedure for validation of defect orientation was introduced (Figure 18).

The diagram of the procedure is presented in Figure 18d. The process was based on the fused results of Stage II obtained for the *DF*_PYR_ rule. First, the statistical analysis was processed and the thresholding procedure was applied. The threshold was adjusted to the mean value of part of the signal for which the level was higher than its 3rd quartile. As a result, the 2-D distribution was obtained, depicting the indicated defected area *DA*. Next, principal component analysis (PCA) was carried out for the achieved spatial distribution of points in the (*l*, *α*) space, allowing one to define two axes of main variation of the *DA*: *PC*_1_ and *PC*_2_. In consequence, the length of the sections defining the dimensions in both main directions were obtained. In order to determine the final length of data variability in the samples axial and circumferential directions, the *PC*_1_ and *PC*_2_ sections were projected into the *l* and *α* axes of the sample. The achieved orientation evaluation results are presented in Table 3. One can notice that in each case, the longer dimension of the defect was properly indicated. The longer dimension in the axial direction was evaluated for defects *d*_1_, *d*_3_, and *d*_5_ (axial defects), and in the circumferential direction for defects *d*_2_, *d*_4_, and *d*_6_ (axial defects). However, it must be pointed that the proposed procedure can be used only for the orientation indication of the longer dimension of defects, as the achieved evaluated values differ largely from the real one. 

**Figure 19:**
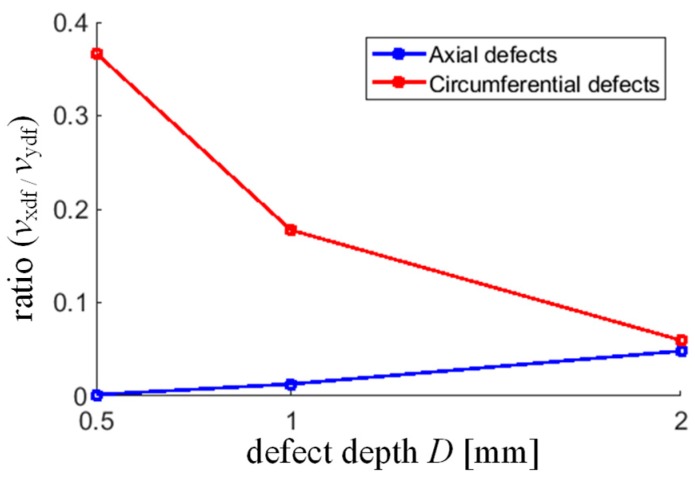
Results of defect depth evaluation based on the ratio of *v*_xdf_ and *v*_ydf_.

Further, taking into consideration the obtained grouping results, the changes in defect signal amplitude were analyzed for both orientations. The results of the ratio of fused results of components *x* and *y* (Stage I) are presented in Figure 19. It can be noticed that there is clearly visible change of the ratio with the growing depth of the defects. Depending on the direction of the defect, the increase in depth causes an increase in the amplitude of a particular component of the vector of field leakage sensed by the array. Therefore, in the case of circumferential defects, the ratio is decreasing and in axial ones, it is growing. Nevertheless, the obtained data confirmed the possibility to utilize the fused results in further processes of precise characterization of defect dimensions.

## 7. Conclusions

In this paper, a multisensor array was utilized to perform data fusion procedures under various measuring conditions, allowing adjusting systems in terms of reliability and inspection time. The study was carried out considering inspection data of defects in a circular-shaped tube sample. During data collection, the vector magnetic leakage field over the area where the defects occurred in the examined steel object was monitored. The observation was carried out for defects with different depths and orientations in reference to the direction of the magnetizing field. The experiments were conducted for various sensor configurations. Next, multisensor data registration was carried out in order to transfer all data into a common space. Then, four multisource signal fusions were introduced and applied for all modes of sensor array configurations. Finally, the quality of the obtained results was assessed. For that need, the entropy and signal-to-noise ratio were computed for all utilized sensor configuration strategies. Next, in order to validate the information content of the data achieved during the fusion process, an additional experiment was carried out to assess the defect indication capability.

It was shown that the application of data fusion rules for a magnetic multisensor inspection system can result in a growth in reliability of the proper identification and classification of defects in steel elements. In accordance with the system operation objective demanded by the end user, a different data fusion algorithm could be implemented. Each sensor in the array defines its own set of data. When considering the character of the repose signal caused by the defect, a common representation can be built by calculation of cumulative magnetic vector lengths. Such an approach was carried out using the *DF*_vec_ algorithm. The other way to build a conjunctive representation, allowing preservation in the fused result of all data supplied by each sensor, the addition operation of set contents can be carried out. Frequently, this methodology is updated with weighting factors corresponding to each set’s importance (in terms of final set definition). In such a case, referred to as the “compromise” representation, the addition operation can be supplemented with the weights calculated on the basis of the coefficients describing the data. In this paper, for this purpose, the *DF*_SF_ algorithm was used. It allows one to simultaneously build a representation of all sensors’ full data in the final result, and also to reward and penalize the sources in terms their data quality level. On the other hand, it must be pointed that both useful information (concerning the defected area) and background signals are transferred into the resulting distribution under this method. Therefore, considering achieving a higher confidence level in the indication of the defect area, the *DF*_IOP_ algorithm was applied. It validates in the final result only the part of the data which is common for all input sources. Thus, the random background signal level is much reduced. On the other hand, even a small error in the registration process can also affect the signal distribution in the defect area. High efficiency in noise reduction when building a compromise representation can be also achieved by the application of multiresolution analysis methodology (*DF*_PYR_ algorithm). It allows one to transform the input data into a series of low or bandpass representations at different frequency bands. Thus, both the low-frequency background signal and the high-frequency noise can be significantly reduced. In the same time, the defected area signal representation remains practically unchanged. The subjective and objective assessment of the fusion results confirmed higher performance of the multisensor system in comparison to single-source data. The signal fusion scheme can be relatively easily applied in industrial measuring systems. Simultaneous use of algorithms *DF*_IOP_ and *DF*_PYR_ can significantly affect the increment of levels of correct indication of the defects. Nevertheless, the obtained results showed that the configuration of multisensor systems should be deeply considered in accordance with multiple aspects.

## Figures and Tables

**Table 1:** Evaluation of data fusion scheme based on entropy *E*.

Name	Entropy					
	*d*_1_	*d*_2_	*d*_3_	*d*_4_	*d*_5_	*d*_6_
Raw Signal	6.98	6.35	7.34	6.81	6.07	6.19
*DF*_vec_	6.98	6.28	7.38	6.77	6.24	6.14
*DF*_SF_	7.00	6.27	7.42	6.84	6.26	6.15
*DF*_IOP_	1.36	0.17	1.73	0.29	0.09	0.20
*DF*_PYR_	6.22	5.11	6.75	5.37	5.38	5.25

**Table 2:** Evaluation of data fusion scheme based on *SNR*.

Name	Signal-to-Noise Ratio (*SNR*) (dB)					
	*d*_1_	*d*_2_	*d*_3_	*d*_4_	*d*_5_	*d*_6_
Raw Signal	8.89	10.66	6.54	10.37	15.09	10.97
*DF*_vec_	9.09	11.44	6.78	11.54	14.85	11.82
*DF*_SF_	8.83	10.44	6.31	10.60	14.06	11.50
*DF*_IOP_	45.79	58.94	44.46	56.62	64.91	59.10
*DF*_PYR_	14.70	21.94	11.66	20.30	19.96	22.19

**Table 3:** Evaluation of inspected defect orientation.

Defect	Estimated Dimension in α Direction (mm)	Estimated Dimension in l Direction (mm)	Real Dimensions in α Direction (mm)	Real Dimensions in l Direction (mm)
*d*_1_	1.12	1.43	0.5	5
*d*_2_	2.78	1.66	5	0.5
*d*_3_	2.55	4.36	0.5	5
*d*_4_	3.94	1.56	5	0.5
*d*_5_	1.85	2.49	0.5	5
*d*_6_	4.64	2.30	5	0.5
